# Understanding the habitat vulnerability of Slums to COVID 19: Case of two megacities of India

**DOI:** 10.12688/f1000research.153809.1

**Published:** 2024-07-30

**Authors:** Sudha Panda, Soumyendu Shankar Ray

**Affiliations:** 1 School of Architecture and Planning, Kalinga Institute of Industrial Technology, Bhubaneswar, Odisha, 751024, India; 2 School of Architecture and Planning, Kalinga Institute of Industrial Technology, Bhubaneswar, Odisha, 751024, India

**Keywords:** Habitat vulnerability; COVID-19; urban slums; congestion; access to basic services; risk exposure;

## Abstract

**Background:**

Urban slums are hotspots of infectious diseases like COVID-19 as was seen in the waves of 2020 and 2021. One of the primary reasons why slums are disproportionately affected is their location in inaccessible and uninhabitable zones, crowded and poorly ventilated living spaces, unsanitary conditions and common facilities (water taps, common toilets, etc.). Staying at home during pandemics is hardly an option for slum dwellers as it often means giving up work and even basic necessities.

**Methodology:**

This paper aims to understand the habitat vulnerabilities of slums in the two Indian megacities of Pune and Surat which were the worst hit during both waves. The study is done at the level of wards, which is the smallest administrative boundary, taking the habitat vulnerability (congestion and access to basic services). To identify the explanatory variables which increase the vulnerability of slums to infectious diseases, literature study is done on the triggering factors which affect habitat vulnerability derived from common characteristics and definitions of slum.

**Results:**

The aim of the research is to categorize the slums into 3 levels of risk zones and map them subsequently.

**Conclusion:**

This study will help in formulating a model to prioritize the allocation of sparse resources in developing countries to tackle the habitat vulnerabilities of the slum dwellers especially during health emergencies of contagious diseases like COVID-19.

## Introduction

India is among the foremost countries which were severely impacted by COVID-19 in the 2021 wave. It managed to dodge the severity of the impact of the 2020 wave by imposing a very rigid lockdown which just about managed to flatten the curve giving the government the time to scale up the public health system. But in future if we are to be better equipped to contain the transmission of a pandemic we have to primarily identify the risk factors that catalyze the disease spread in our urban realm and take appropriate steps to counter the same. It was observed that states with greater urban population, relatively higher population density, greater slum population, and a comparitively higher crowding in dwellings had a higher incidence of COVID-19. This was further intensified by comparatively poor medical facilities and lower expenditure on health infrastructure ( Pathak et al., 2020). Social distancing which reduce exposure to COVID 19 is difficult to practice in slums which have crowded and congested living ( Corburn et al., 2020; Friesen and Pelz, 2020). Besides space restriction and congestion, the lack of basic amenities like water supply, toilets, sewerage system, drainage facilities, solid waste collection, secure and adequate housing and existing poor medical conditions of the slum dwellers further reinforces their vulnerability to COVID-19 ( Khatua, 2020).

The objective of this research is to obtain a critical understanding of the relationship between habitat vulnerability, health and slums so that risk exposure of slum dwellers can be quantified. The study is done at the smallest administrative level i.e. at ward level so that it is easy to identify the susceptible wards in a pandemic, enabling greater resources to be diverted to these places and taking preventive steps to counter the risk factors.

### Background

Spread of the pandemic in urban centers was a global phenomenon. Cities with a larger population had a larger infection base and India was no exception with almost 40% of the cases in the 2020 wave being found in the 4 major metro cities of Delhi, Mumbai, Kolkata and Chennai which see a lot of international visitors. But the rapid spread of the contagion there onwards mainly happened where the urban concentration was more. The Census 2011 report states that one-third of India’s population lives in slums. Considering that the highly congested slums are breeding grounds for COVID 19 which thrives on proximity, it is imperative to understand the inherent habitat vulnerability of these slums to be able to propose an effective and long term policy planning to reduce these risk factors. Research has shown that in Mumbai, settlements have been built around industrial areas like garment factories where people stay in cramped spaces with eight people sharing a room surrounded by hazardous chemicals and machines ( Parpiani M., 2020).

Daily wage laborers and migrant labor constitute a major chunk of the slum dwellers ( Ghosh et al., 2020). Social distancing is a safeguard against the spread of COVID-19 but it is a luxury which cannot be afforded by daily wage laborers living in slums ( Courtemanche et al., 2020; Weill et al., 2020). For most slum dwellers it is important to continue livelihood activities which will ensure an income and subsequently food security than the possibility of contracting COVID-19.

### Literature study

To have a better understanding of the relationship between ‘Slums’, ‘habitat vulnerability’ and ‘health’ it is important to delve deeper into their definitions given by noted organizations.

*Slums*

The term “slum” refers to squalid housing within urban areas that are overcrowded and have inadequate provision of basic services. UN-HABITAT definition for a slum household needs to satisfy one or more of the five conditions given below:

- Temporary housing that does not give protection against extreme climate conditions;
- Inadequate dwelling space;
- Availability of affordable, sufficient and safe water;
- Provision of a private or public toilet and
- Security of tenure ( UN-Habitat 2003a; 2003b; 2006/7).

World Health Organization (WHO) recommends 2m physical distancing as a preventive against COVID 19 spread which is next to impossible in a slum household where 5-6 persons are packed in a 25-30 square meters dwelling with poor ventilation which is further aggravated due to cooking methods which rely on coal or wood as fuel. Hygiene and access to clean water is a critical parameter for precaution against COVID-19 but with large number of people dependent on municipality tankers for water and very low ratio of private toilets it is very difficult to practice the hand washing and hygiene norms suggested by WHO.

We take a comparative look in Table 1 on various definitions and parameters (Legality, Density, Housing, Water and Sanitation) by various noted national and international organizations to understand their commonality.

**Table 1.** Parameters of Slum definition by various organizations.

Organisations	Legality	Density	Housing	Water	Sanitation
**Census**	Recognized as Slum by State/Local Govt under any Act including Slum Act	Should have a population of around 300 persons	Temporary overcrowded settlements in usually unsanitary conditions with lack of infrastructure	Inadequate potable water supply	Lacking proper sanitation facilities
**National Family Health Survey**	NA	Should have a population of around 300 persons	Temporary overcrowded settlements in usually unsanitary conditions with lack of infrastructure	Inadequate potable water supply	Lacking proper sanitation facilities
**UN Habitat**	NA	Inadequate dwelling space	Temporary housing that does not give protection against extreme climate conditions	Availability of affordable, sufficient and safe water	Lack of access to improved sanitation facilities

Source: UN-Habitat, 2003a, 2003b, 2006/7; Gupta et al., 2009 (NFHS); Ministry of Housing and Urban Poverty alleviation, 2008.

*Vulnerability and risk exposure*

The term “Vulnerability” refers to certain inherent and inbuilt set of conditions that maintain people in a perilous and hazardous conditions. In the context of susceptibility to health, it implies situations leading to risk to diseases. It can also be defined as *“the degree to which a system, subsystem or system component is likely to experience harm due to exposure to a hazard, either to a perturbation or stress or stressors*” ( Turner et al., 2003). Risk is the chance (or possibility) that the hazard will reoccur. Vulnerability is a function of exposure, sensitivity and adaptive capacity of the system ( Cutter, 1996).

*Health vulnerability of Slums to COVID 19 due to habitat conditions*

The definition of vulnerability in the context of health leads us to understand the inherent structural conditions in slums that makes the slum dwellers prone to contagious diseases like COVID-19. It mainly spreads through contact, hence the health vulnerability in slums is mainly due to the inability to practice physical distancing and other conditions which aid the disease transmission once the disease has been contracted. The literature study revealed that the health vulnerability parameters which exist in slums can be clubbed into two types of factors

1. Socio Economic Conditions
2. Habitat Condition

Table 2 (below), clubs all the factors derived from literature study into the above headings to later filter out the factors can be controlled or improved.

**Table 2.** Parameters impacting health vulnerability of slums to COVID-19.

	Factors	Themes	Parameters	Sources
HEALTH VULNERABILITY FACTORS IN SLUMS	Factors preventing **Social Isolation**	**Socio Economic Conditions**	Working for survival-daily wage labor	1. Ghosh et al., 2020. 2. Khatua, 2020. 3. Courtemanche et al., 2020. 4. Weill et al., 2020.
			Mostly migrant labor	1. Ghosh et al., 2020. 2. Khatua, 2020 3. Courtemanche et al., 2020.
			Low level of education so less disease awareness	1. Pathak et al., 2020.
			Joint and extended families with 7-8 people in cramped quarters	1. Pathak et al., 2020.
			Asymptomatic nature of disease	1. George et al., 2021.
			Fear and stigma associated with disease	1. George et al., 2021.
			Mistrust between slum dwellers and health officers	1. George et al., 2021.
		**Habitat conditions**	High Population	1. Sclar et al., 2005. 2. Gibson and Rush, 2020. 3. Wasdani and Prasad, 2020.
			High Density	1. Corburn et al., 2020 2. Gibson and Rush, 2020. 3. Wasdani and Prasad, 2020.
			Illegality of settlements	1. Ayeb-Karlsson, 2020. 2. Ayeb-Karlsson et al., 2020. 3. Ezeh et al., 2017.
			Very less ventilation and congestion	1. Corburn et al., 2020.
			Erratic electric supply coupled with low ventilation	1. Raju, 2020.
			Multipurpose business related use of household	1. Pathak et al., 2020.
			Most products hand related so isolation difficult	1. Pathak et al., 2020.
			Narrow slum pathways making medical help difficult	1. Raju and Ayeb-Karlsson, 2020.
	Factors facilitating **disease transmission**	**Socio Economic Conditions**	Inadequate data availability on number, living condition, and health comorbidity	1. Friesen and Pelz, 2020. 2. Pathak et al., 2020
			Lower health expenditure by family	1. Pathak et al., 2020.
		**Habitat conditions**	Inadequate access to sanitation, waste disposal and healthcare facilities	1. Corburn et al., 2020. 2. Ghosh et al., 2020. 3. Sclar, Garau and Carolini, 2005. 4. Subbaraman et al., 2012.
			Most slums are not notified, hence excluded from basic amenities	1. Nolan et al., 2017.
			Drinking water at a distance from homes	1. Raju et al., 2021. 2. Raju, 2020.
			Poor health infrastructure	1. Raju et al., 2021. 2. Raju, 2020.
			Continuous handwashing not possible (lack of water and soap)	Raju and Ayeb-Karlsson, 2020.

## Methods

The study was conducted with an objective to understand the existing habitat vulnerability which is present in slums which aggravates the susceptibility of the slum dwellers to contagious diseases like COVID-19. In order to identify these explanatory variables, the characteristics which define a slum given by various organizations are studied and superimposed on the habitat and socio-economic factors identified by previous literature which are considered responsible for increasing the vulnerability of slum dwellers. Data is collected about the common parameters (congestion and access to basic services) from sample cities of Surat and Pune wherein a ward (smallest administrative border in the city) is the unit of comparison.

**Figure 1.**
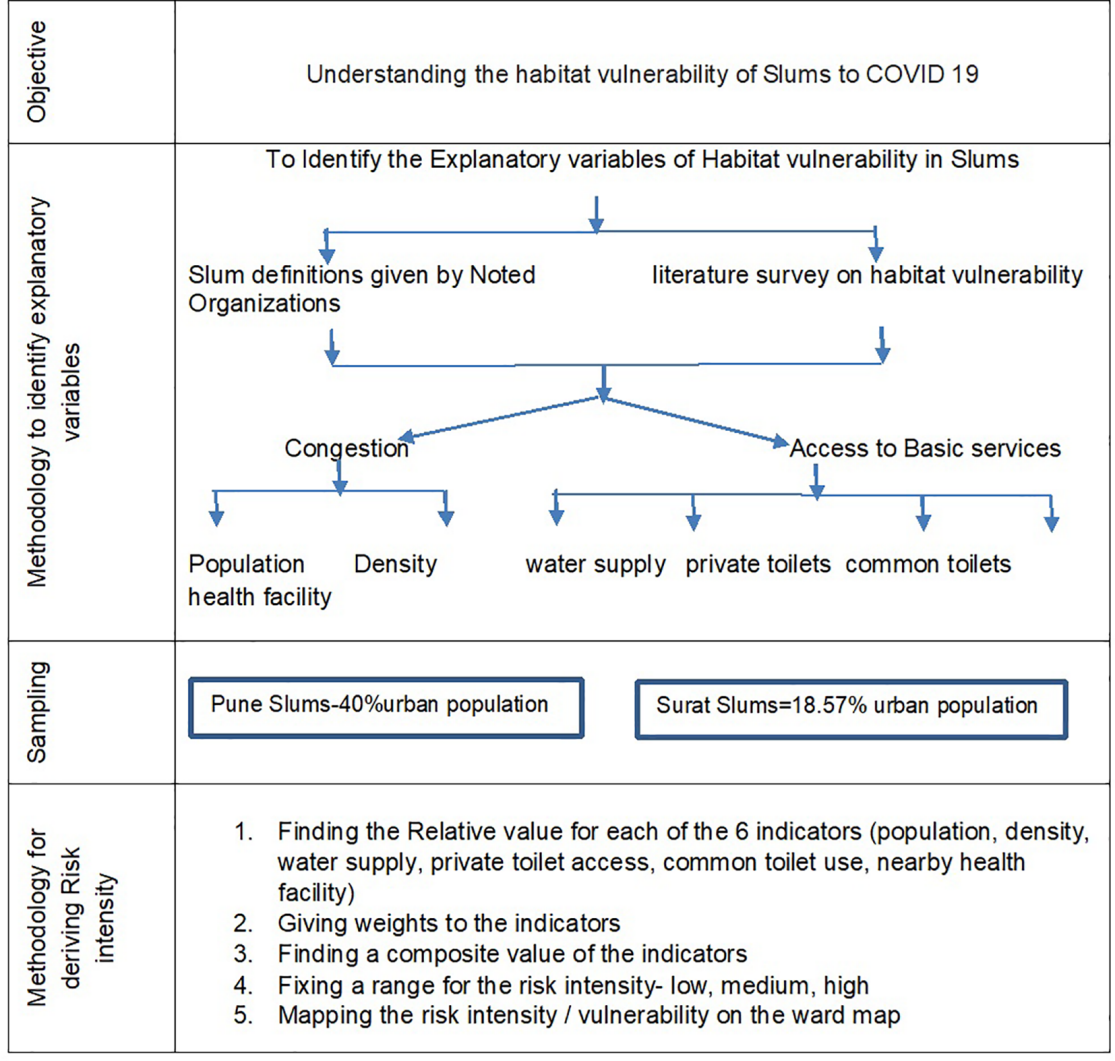
Steps in the research process.

To derive the risk intensity, the raw data is transformed into relative values to normalize all values so that they range from 0 to 1. After giving weights to the indicators a linear summation is done to obtain a composite value for the risk exposure and depending on the range classified into high, medium or low. When this risk exposure value is referenced on the ward map of the city, a lucid and graphic picture emerges for policy planners while prioritizing the allocation of scarce resources to areas which demand urgent attention and to be fully prepared in the event of a disaster like COVID-19. Figure 1 (below) shows the complete research steps followed to achieve this end.

### Sampling

Pune and Surat have been selected for testing this model because they are contrasting cases of cities in the way they have dealt with slum issue. Pune has 40.56% of its population living in slums, whereas Surat has only 19.24% of its population living in the slums. The low slum population in Surat is due to the aggressive implementation of slum re-location and development programs after the plague in 1992.

*Pune*

Pune municipal corporation (PMC) is responsible for the provision of basic services in slums under the Maharashtra Slum Improvement and Clearance Act of 1971. As of 2011, Ghole Road, Dhole Patil Road and Sangamwadi ward shows the maximum percentage of slum population (between 40-50%). The least number of slums population is found in Bibvewadi, Kasba and Dhankawadi wards (less then 5%). Tilak Road ward and Karve Road have maximum number of declared slum i.e. about 13% and 12% respectively.

**Figure 2.**
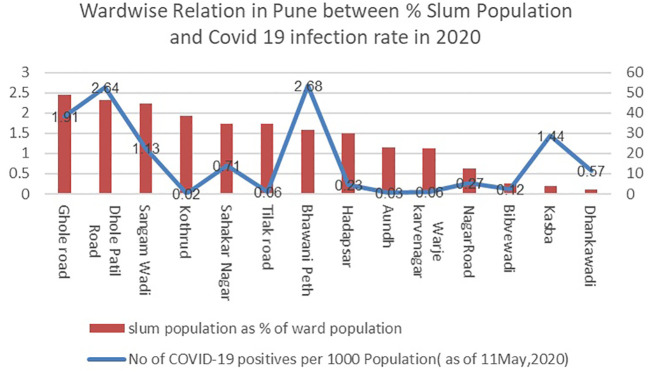
Wardwise relation in Pune between % Slum Population and Covid 19 infection rate in 2020. (Source: Pune Municipal Corporation, 2020).

**Figure 3.**
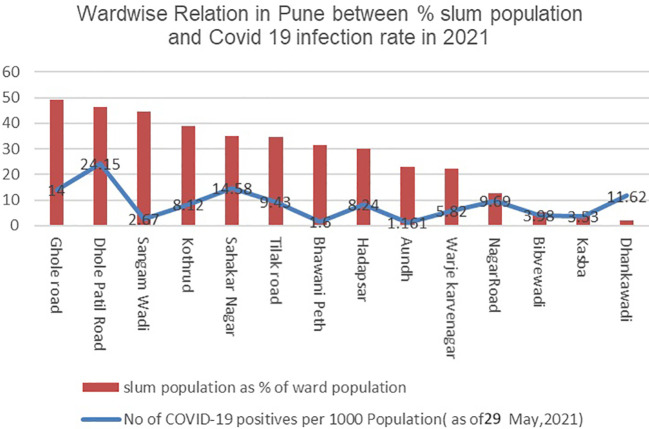
Wardwise relation in Pune between % Slum Population and Covid 19 infection rate in 2021. (Source: Mohol, 2021).

A study of the trend between slum population percentage and COVID-19 infection rate during both waves of 2020 and 2021(as seen in Figures 2 and 3) shows that there’s no particular relationship that would imply a higher population in slums having a higher infection rate.

*Surat*

Slums in Surat constituted 27.5 percent of the city’s population in 1992. However, after 1992, the slum growth rate has decreased from an annual average of 14.6 percent in 1992 to an annual average of 1.46 percent in 2001.

**Figure 4.**
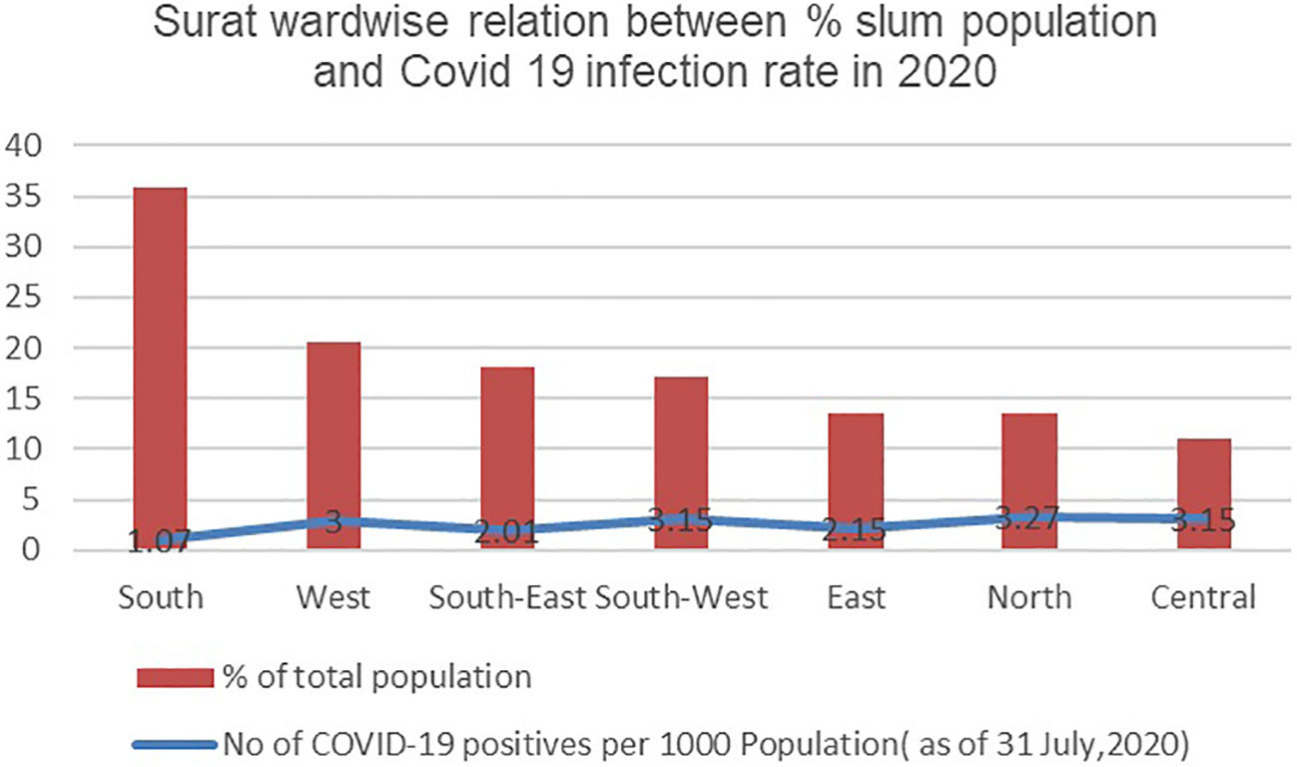
Wardwise relation in Surat between % Slum Population and Covid 19 infection rate in 2020. (Source: Surat Municipal Corporation, 2020).

**Figure 5.**
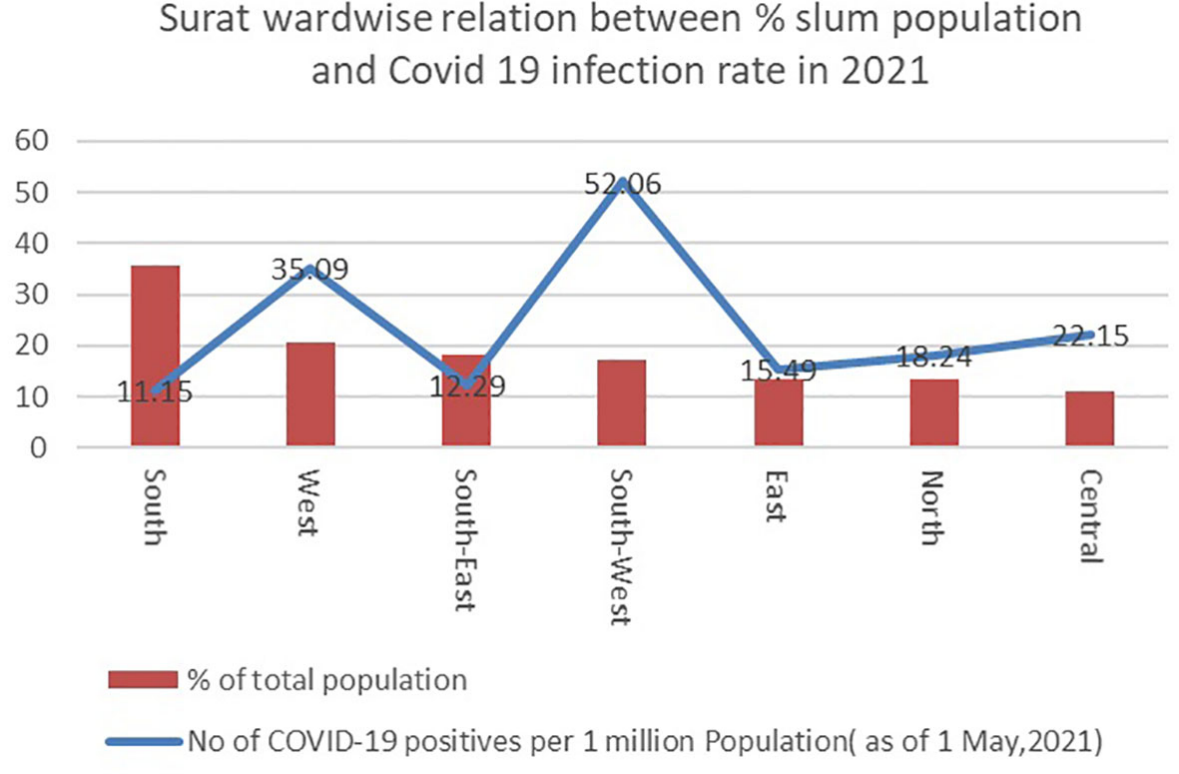
Wardwise relation in Surat between % Slum Population and Covid 19 infection rate in 2021. (Source: Surat Municipal Corporation, 2021).

In Surat too, a study of the trend between slum population percentage and COVID-19 infection rate during both waves of 2020 and 2021(as seen in Figures 4 and 5) shows the same results as in Pune. There’s no particular relationship that would imply a higher population in slums having a higher infection rate.

*Empirical Model*

Based on the habitat vulnerability parameters obtained from the literature study and superimposing on the parameters which define a slum (given by noted organizations) it was seen that ‘Congestion’ and ‘Lack of access to Basic Services’ come out as the most significant parameters responsible which create vulnerability of slums to COVID-19. The two indicators selected under ‘Congestion’ were ‘Population’ and ‘Density’ (persons per unit square km in slums). The four indicators selected under ‘Lack of access to Basic Services’ were ‘Percentage population with access to water supply’, ‘Percentage population using private toilet’, ‘Percentage population using common toilet’ and ‘Percentage population with near accessibility to health facility’. Expert opinion on the weightage which should be given to the final six indicators were 0.25 to each of the two ‘Congestion indicators’ and 0.125 to each of the four ‘Lack of access to Basic Services’ indicators.

Hence the linear model obtained was

Risk Exposure of a slum = *α*1 + *β*1 ***Congestion factors*** ( *f*(‘Population’ *,* ‘Density’)) + ***Lack of access to Basic services*** ( *f*(‘Percentage population with access to water supply’, ‘Percentage population using private toilet’, ‘Percentage population using common toilet’ and ‘Percentage population with near accessibility to health facility’)) + *εi*

### Data collection

True values of indicators for sample cities of Pune and Surat are seen in Tables 3 and 4 (below)

**Table 3.** True values for indicators of Pune slums.

Ward	Congestion		Access to basic services			
	Slum population	Population density/sqkm of slum	% population with access to water supply	% population using private toilet	% population using common toilet	% population with near accessibility to health facility
Aundh	41475	151923	61.6	12	33.3	73
Kothrud	81045	170621	67.9	45	21.7	77
Ghole road	84405	187984	72.8	25	32.3	77
Warje karvenagar	52245	120658	62.3	23	20.4	66
Dhole Patil Road	72040	140703	64.1	18	17.9	76
Hadapsar	84465	117476	62.9	18	7.5	73
NagarRoad	29775	145956	80.3	31	19.6	84
Sangam Wadi	116390	135495	66	52	29.4	74
Bhawani Peth	60615	206174	74.9	14	34.5	75
Kasba	8880	17976	73.9	1	16.9	76
Sahakar Nagar	70900	212275	64.7	22	15.6	77
Tilak road	83595	154177	62.1	22	16.7	74
Bibvewadi	15725	201603	65.1	10	12.3	73
Dhankawadi	5260	228696	69.4	14	20.8	69

Source: Mahajan S, 2010. *Housing Study for Pune Municipal Corporation (2009-2010).*

**Table 4.** True values for indicators of Surat slums.

Ward area	Congestion		Access to basic services			
	Population	Population density/sqkm of slum	% population with access to water supply	% population using private toilet	% population using common toilet	% population with near accessibility to health facility
East	711516	252405	42	4	34	56
West	449943	112888	97	37	75	20
North	716110	293287	59	7	62	8
South	881070	229663	68	10	60	26
Central	408760	77213	68	58	38	100
South-West	348423	150036	70	64	57	70
South-East	754128	172494	77	62	49	51

Source: Surat Urban Development Authority (2006-12).

## Results and discussion

As can be seen in the linear model above measuring risk exposure of the slums the indicators for “congestion” and “access to basic services” have different measurement units (persons, persons/sqkm and percentages etc). However, to make these indicators comparable they need to be transformed into a standard form. Hence, each of the indicators included in the analysis, is normalized using the actual, minimum and maximum risk threshold values. The value of the normalized relative indicator varies between 0 to 1 and is calculated thus:

$$\text{Relative indicator}=\frac{\text{Actual value}–\text{Minimum value}}{\text{Maximum value}–\text{Minimum value}}$$ (1)

For some indicators, a higher score is equivalent to a higher risk (as in the congestion factors), whereas for other indicators, a higher score might imply lower risk (as in percentage access to water and individual toilet facilities). For the scores to be formulated according to higher the value the lower the risk (as in percentage access to water and individual toilet facilities), the values are transformed into negative values.

To obtain a composite value of the risk

$$\mathrm{Ri}=\sum_{k=1}^{l}w_{k}\;x_{\mathrm{ik}}=w_{1}\;s_{\text{i1}}+w_{2}\;s_{\text{i2}}++\ldots \ldots .w_{m}\;s_{\mathrm{il}}$$ (2)

Where *Ri* the overall score of Risk *i* and *S _il_* the Relative indicator value *i* for criterion *j* of which *wj* is the weight.

Calculating the composite values of the Risk exposure across the different wards in Pune and Surat, the following values are obtained as seen in Tables 5 and 6.

**Table 5.** Risk exposure of slums in different wards of Pune.

Ward	Relative value of population	Relative value of population density	Relative value of %population with access to water supply	Relative value of %population using private toilet	Relative value of %population using common toilet	Relative value of %population with near accessibility to health facility	Composite risk value	Risk range
Aundh	0.325	0.635	0	0.25	1	0.389	0.285	High
Kothrud	0.681	0.724	0.336	1	0.55	0.611	0.176	Medium
Ghole road	0.712	0.806	0.598	0.545	0.961	0.611	0.280	High
Warje karvenagar	0.422	0.487	0.037	0.5	0.5	0	0.222	High
Dhole Patil Road	0.600	0.582	0.133	0.386	0.403	0.556	0.211	High
Hadapsar	0.712	0.472	0.069	0.386	0	0.389	0.190	Medium
Nagar Road	0.220	0.607	1	0.682	0.469	1	-0.069	Low
Sangam Wadi	1	0.557	0.235	1.159	0.849	0.444	0.265	High
Bhawani Peth	0.498	0.893	0.711	0.295	1.047	0.5	0.290	High
Kasba	0.032	0	0.657	0	0.364	0.556	-0.098	Low
Sahakar Nagar	0.590	0.922	0.165	0.477	0.314	0.611	0.260	High
Tilak road	0.704	0.646	0.026	0.477	0.357	0.444	0.264	High
Bibvewadi	0.094	0.871	0.187	0.205	0.186	0.389	0.167	Medium
Dhankawadi	0	1	0.417	0.295	0.516	0.167	0.204	High

**Table 6.** Risk exposure of slums in different wards of Surat.

Ward area	Relative value of population	Relative value of population density	Relative value of %population with access to water supply	Relative value of %population using private toilet	Relative value of %population using common toilet	Relative value of %population with near accessibility to health facility	Composite risk value	Risk range
East	0.682	0.811	0	0	0	0.522	0.308	High
West	0.191	0.165	1	0.55	1	0.13	0.004	Medium
North	0.69	1	0.309	0.05	0.683	0	0.463	High
South	1	0.706	0.473	0.1	0.634	0.196	0.410	High
Central	0.113	0	0.473	0.9	0.098	1	-0.256	Low
South-West	0	0.337	0.509	1	0.561	0.674	-0.119	Low
South-East	0.762	0.441	0.636	0.967	0.366	0.467	0.088	Medium

**Figure 6.**
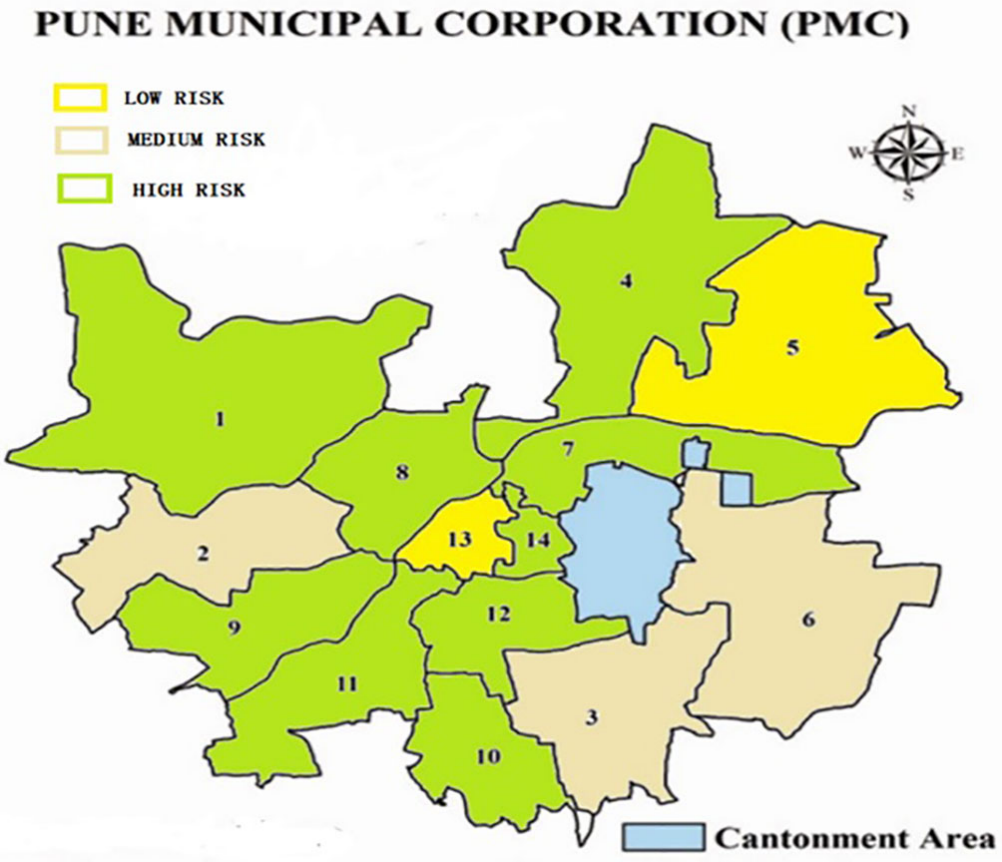
Risk exposure of slums in Pune to COVID 19. (Source: Authors, 2023).

Transforming these numeric values into a more comprehensive picture, a range of > 0.20 is taken as high, 0 to 0.20 is taken as medium and less than 0 is taken as low. Finally, to represent the values graphically, it is graphically represented on the ward map of the cities as seen in Figures 6 (Pune) and 7 (Surat).

**Figure 7.**
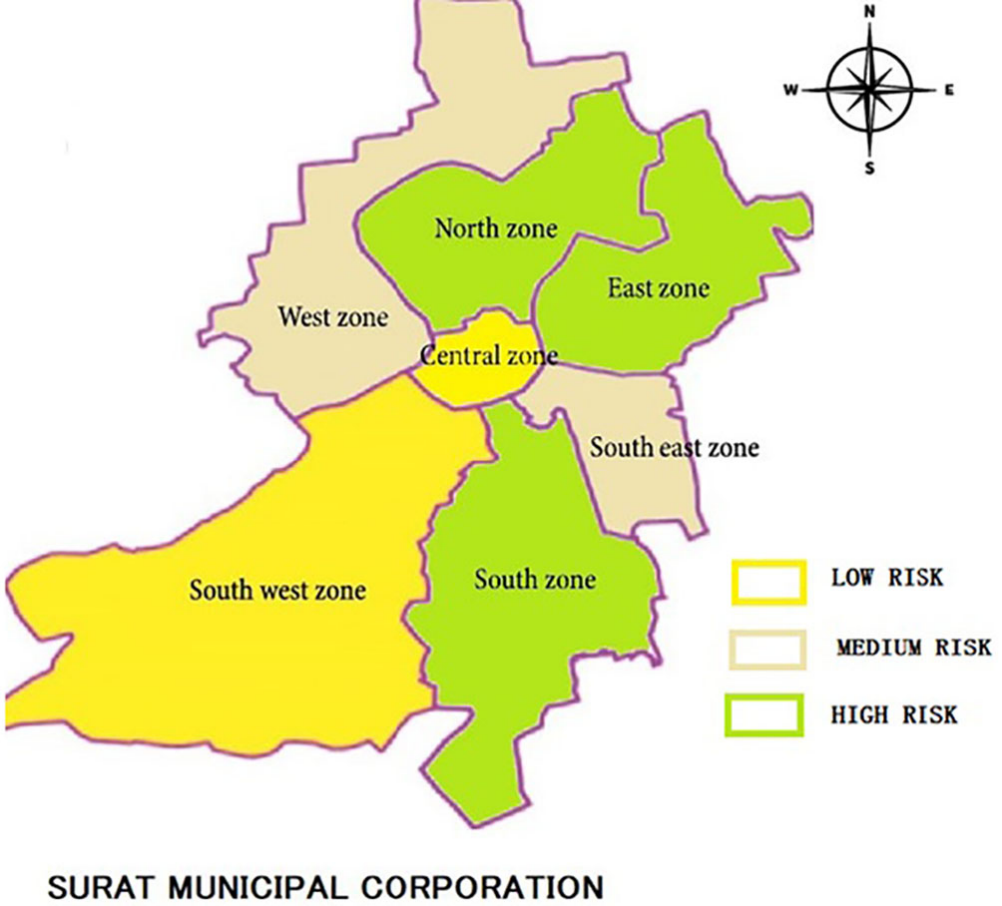
Risk exposure of slums in Surat to COVID 19. (Source: Authors, 2023).

A major part (9 out 15 wards) of the Pune slums fall in the high risk zone. Only 3 are in the medium risk zone and 2 are in the low risk zone. With almost 40.56% of the Pune population living in slums, this spells trouble for the entire city as slums are the hotspots from where the contagion starts spreading.

The picture is much brighter in Surat, in spite of the rapid influx of migrants, as only 3 wards are in high risk zone. This is due to the proactive measures taken by Surat Municipal Corporation (SMC) in 1994 when they took aggressive and rigorous measures after the 1994 plague. However, in spite of the concentrated efforts of the Surat municipality there were open areas in the south and south eastern parts of the city where due to rural migration slums developed again.

## Conclusion

The aim of the paper was to find a method to quantify the risk exposure to pandemics in slums through a critical understanding of the relationship between habitat vulnerability, health and slums which was done by taking a case study of 2 cities of Pune and Surat during the COVID-19 waves of 2020 and 2021. By concentrating on the parameters which define a slum and are simultaneously the triggering factors for the spread of a pandemic (as revealed by the literature study), a method is devised to quantify the risk exposure.

It is important to find both immediate and long term solutions to the problem. COVID 19 has cemented the fact this will not be the first or last contagious disease which the world will witness in a long time but we need to be better prepared to contain such pandemics and the first step lies in curtailing its spread in the hotbeds i.e. the slums of urban centers. By identifying the critical slums the study has found a method for quantifying the risk exposure due to habitat conditions which will help to prioritize the allocation of scarce resources in emergency times and also for long term slum redevelopment planning.

As an immediate measure, access to provision of basic amenities should be prioritized in the times of pandemics by water tanks, mobile sanitizing centers/hand washing stations, portable toilets at critical points which can be done under Swachh Bharat Abhiyaan or Clean India Mission. To incentivize the collection of solid waste, youth in the slum can be encouraged by giving a bag of groceries in exchange for a bag of solid waste. The bags of solid waste obtained can be recycled by the slum dwellers and be a source of employment generation. This can be managed by slum emergency planning committees which will also provide mobile health clinics to carry out testing, diagnosis and immediate treatment. These mobile clinics should be able to maneuver through the existing narrow streets in the slums and be well equipped with equipment for the treatment of COVID-19 and common ailments in urban slums, which are usually diarrhea and respiratory infections. The long term measures should be relocation or redevelopment of slums in a phased manner providing well ventilated housing having all the basic amenities like water, sanitation, drainage, toilets, access to health facilities.

### Informed consent statement

The University has granted consent to participate and publish.

## Author’s contribution

**Panda S**: Conceptualization, Data Curation, Formal Analysis, Investigation, Methodology, Resources, Supervision, Validation, Visualization, writing – Original Draft Preparation, writing – Review & Editing; **Ray SS**: Conceptualization, Data Curation, Formal Analysis, Investigation, Methodology, Resources, Supervision, Validation, Visualization, writing – Original Draft Preparation, Writing – Review & Editing;

## Ethical approval and consent for third party data

The Ethics Committee of the School of Architecture and Planning, KIIT University on 6th June,2024 waived the need for ethics approval and the need to obtain consent for the collection, analysis and publication of the data as it has been obtained from freely available online content which has been properly referenced and cited.

## Consent to participate

All authors have given consent to participate.

## Consent to publish

All matter in paper is original work and not published elsewhere. There is consent to publish.

## Data Availability

1. Figshare: Ward wise Covid 19 Positive Cases in Pune City on May 29, 2021. https://doi.org/10.6084/m9.figshare.26196443 ( Panda & Ray, 2024a). The project contains the following underlying data:
•
Figure 1.jpeg (Covid 19 positive cases in Pune on May 29, 2021). Data are available under the terms of the Creative Commons Attribution 4.0 International license (CC-BY 4.0). 2. Figshare: Ward wise Covid 19 Positive Cases in Pune City on 11 May, 2020. https://doi.org/10.6084/m9.figshare.26196449 ( Panda & Ray, 2024b). The project contains the following underlying data:
•
Figure 2.jpeg (Covid 19 positive cases in Pune on May 11, 2020). Data are available under the terms of the Creative Commons Attribution 4.0 International license (CC-BY 4.0). 3. Figshare: Ward wise Covid 19 Positive Cases in Surat City on 1 May, 2021. https://doi.org/10.6084/m9.figshare.26196455 ( Panda & Ray, 2024c). The project contains the following underlying data:
•
Figure 3.jpeg (Covid 19 positive cases in Surat on May 1, 2021). Data are available under the terms of the Creative Commons Attribution 4.0 International license (CC-BY 4.0). 4. Figshare: Ward wise Covid 19 Positive Cases in Surat City on 31 July, 2020. https://doi.org/10.6084/m9.figshare.26196464 ( Panda & Ray, 2024d). The project contains the following underlying data:
•
Figure 3.jpeg (Covid 19 positive cases in Surat on July 31, 2020). Data are available under the terms of the Creative Commons Attribution 4.0 International license (CC-BY 4.0).
