# Supplementary material for: Understanding the habitat vulnerability of Slums to COVID 19: Case of two megacities of India
Source: F1000Res. 2024 Jul 30;13:849. [Version 1] doi: 10.12688/f1000research.153809.1 (PMC11514380; doi:10.12688/f1000research.153809.1)
Supplement: Peer review history

# Reviewer report: Reviewer response for version 1

**DOI:** 10.5256/f1000research.168749.r326369

**Published:** 2024-10-23

**Authors:** Deepashree Choudhury

**Affiliations:** 1 Apeejay Institute of Technology, Greater Noida, Uttar Pradesh, India

The article investigates the vulnerability of slum habitats to COVID-19 in two Indian megacities. It seeks to understand how the unique living conditions in these informal settlements increase exposure to the virus and influence the overall pandemic response. The study addresses an important topic, given the disproportionate impact of the pandemic on marginalized urban populations. However, while the research is insightful, there are a few  areas where the methodology, data presentation, and discussion could be strengthened to ensure clarity and replicability.

 1. Presentation and Literature Citations

  The paper is well-written, but certain sections, particularly in the introduction and conclusion, could be enhanced by integrating more recent studies on the impact of COVID-19 on slum populations globally and within India. The discussion of the slum environment in relation to pandemic spread is critical.

 Suggestions:

 (i)Strengthen the introduction by adding more recent studies on slum vulnerability to infectious diseases and specifically COVID-19.

 (ii)Provide proper citations for claims related to the broader impacts of COVID-19 on slums beyond the two cities studied.

 (iii)Clarify how this study builds upon or diverges from other research in this domain.

 2. Study Design and Technical Soundness

 The study provides valuable insights, but the rationale for choosing these two specific megacities (Pune and Surat) needs to be expanded. The authors only say’ Pune and Surat have been selected for testing this model because they are contrasting cases of cities in the way they have dealt with slum issues. Pune has 40.56% of its population living in slums, whereas Surat has only 19.24% of its population living in the slums. The low slum population in Surat is due to the aggressive implementation of slum re-location and development programs after the plague in 1992.‘   The authors do not fully explain whether the findings can be generalized to slums across India or if they are unique to these particular cities.

  Suggestions:

 (i)  Add more robust justification as to why Pune and Surat were chosen as case studies over other cities that also have significant slum populations, such as Kolkata or Chennai.

 (ii)Discuss whether these findings are representative of slum vulnerability across India or only applicable to the megacities studied.

 3. Details of Methods and Analysis

 Providing a clear breakdown of how data was collected and Specifying the criteria used to select slums in these cities for the analysis will help enrich this research. A few questions come to the mind of the reader like, whether any specific sampling method was used, and whether the research involved any first-hand data collection(through interviews with slum dwellers or government officials) ? Were the selected case studies representative of slums across the megacities?

 4. Statistical Analysis and Interpretation

 (i)The authors state that expert opinions were used to assign weightage to the final six indicators—0.25 for each of the two ‘Congestion indicators’ and 0.125 for each of the four ‘Lack of Access to Basic Services’ indicators. However,an elaboration on how this expert opinion was collected (the process and criteria used to select experts, as well as the methods employed (e.g., surveys, interviews, consensus-building techniques) will highly aid  in making the article more comprehensible and in  validating the weightage assigned to the indicators.

 (ii) The authors mention that numeric values were transformed into categories (high, medium, low) based on specific ranges. Detailing out how the risk exposure benchmarks were derived,  for the ranges designated as high, medium, and low will help in establishing the validity of the conclusions drawn.

 (iii)    In Tables 5 and 6, it is suggested that indicators be assigned nomenclature (e.g., Si1, Si2, etc.) to facilitate clearer comprehension and reference throughout the text. This labeling would enhance the readability of the tables and make it easier for readers to track the indicators discussed.

 5. Conclusions

 While the conclusions align with the overall findings, they are somewhat overstated, particularly regarding the implications for policy. The authors suggest wide-ranging solutions to slum vulnerability, but these are not fully supported by the data presented. The discussion would benefit from a more cautious interpretation of the findings, especially given the limitations of the study.

 Suggestions:

 (i)Acknowledge the limitations of the study more explicitly in the conclusions, especially in terms of data limitations and the representativeness of the findings. Provide more grounded, evidence-based policy recommendations that are supported by the specific data from the study.

 (ii)Consider offering suggestions for future research that could address gaps identified in this study, particularly in terms of broader applicability to other cities and long-term vulnerability assessment.

 (iii)separate out recommendations from the conclusion section and present them under a different head.

Is the work clearly and accurately presented and does it cite the current literature?

Partly

If applicable, is the statistical analysis and its interpretation appropriate?

Yes

Are all the source data underlying the results available to ensure full reproducibility?

Yes

Is the study design appropriate and is the work technically sound?

Partly

Are the conclusions drawn adequately supported by the results?

Partly

Are sufficient details of methods and analysis provided to allow replication by others?

Partly

Reviewer Expertise:

Built Environment,Architecture,Urban Design,Urban Studies,Research methodology

I confirm that I have read this submission and believe that I have an appropriate level of expertise to confirm that it is of an acceptable scientific standard.

## References

[1] : Case Selection Techniques in Case Study Research. *Political Research Quarterly* .2008;61(2) : 10.1177/1065912907313077 294-308 10.1177/1065912907313077

[2] : On the Methodological Framework of Composite Indices: A Review of the Issues of Weighting, Aggregation, and Robustness. *Social Indicators Research* .2019;141(1) : 10.1007/s11205-017-1832-9 61-94 10.1007/s11205-017-1832-9

# Reviewer report: Reviewer response for version 1

**DOI:** 10.5256/f1000research.168749.r326376

**Published:** 2024-09-28

**Authors:** Partha Sarathi Mishra

**Affiliations:** 1 Sri Sri University, Cuttack, Odisha, India

Summary:

 This paper makes a valuable contribution by examining the habitat vulnerability of slums to COVID-19 in two major Indian cities, Pune, and Surat. The authors develop a quantitative risk assessment model based on congestion and access to basic services to categorize slum areas into high, medium, and low risk zones. The visual mapping of these risk zones provides an intuitive understanding of vulnerability patterns, which can inform targeted interventions.

 Major strengths:

 - Addresses a critical and timely topic of slum vulnerability to pandemics

 - Develops a quantitative risk assessment model that can be applied to other urban areas

 - Provides clear visual representation of risk zones through ward-level mapping 

 - Offers both immediate and long-term recommendations for reducing vulnerability

 - Uses data from two different cities to demonstrate broader applicability of the approach

 Minor revisions suggested:

 1. Consider including a brief overview of Indian definitions of slums to complement the international definitions provided. This would strengthen the contextual relevance of the study.

 2. Provide some additional background on why Pune and Surat were selected as case studies, perhaps with a sentence or two of data on their COVID-19 impacts.

 3. Add a few more details on data collection methods and sources in the methodology section to enhance reproducibility.

 4. Ensure consistency in how ward data is presented across all tables and figures for clarity.

 5. Simple Additive Weighting Method is mentioned in this paper. However, the weighting method and their implications are not available in this paper. Please clarify on this issue

 6. Briefly explain the rationale for the risk score thresholds used (high >0.20, medium 0-0.20, low <0) to help readers interpret the results.

 7. Consider adding a short limitations section to acknowledge any constraints of the study.

Is the work clearly and accurately presented and does it cite the current literature?

Yes

If applicable, is the statistical analysis and its interpretation appropriate?

Partly

Are all the source data underlying the results available to ensure full reproducibility?

Partly

Is the study design appropriate and is the work technically sound?

Yes

Are the conclusions drawn adequately supported by the results?

Partly

Are sufficient details of methods and analysis provided to allow replication by others?

Partly

Reviewer Expertise:

Architectural Design; Heritage Assessment; Statistical Analysis in Architecture and Planning, Multi-Criteria Decision- Making Analysis

I confirm that I have read this submission and believe that I have an appropriate level of expertise to confirm that it is of an acceptable scientific standard, however I have significant reservations, as outlined above.

# Reviewer report: Reviewer response for version 1

**DOI:** 10.5256/f1000research.168749.r314475

**Published:** 2024-08-29

**Authors:** Natraj Kranthi

**Affiliations:** 1 SPA Bhopal, Bhopal, Madhya Pradesh, India

1. Summary of the Paper - The paper titled ‘Understanding the habitat vulnerability of Slums to Covid 19: Case of two megacities of India’ aims to categorize the slums into three levels of risk zones for mapping. For this purpose, ward wise habitat vulnerabilities of slums in the two Indian megacities of Pune and Surat were analysed. Definitions given by UN-Habitat and WHO were reviewed and vulnerability parameters ‘congestion’ with two indicators and ‘lack of access to basic services’ with four indicators were identified. Risk exposure was measured based on the normalized true values. Risk was calculated and categorized as high (>0.2), medium (0-0.2) and low (<0). Accordingly these ward wise risks maps were done. The paper concludes by suggesting the importance for immediate and long term solutions
2. Observations

- i. Definitions - The definitions of slums given by the international organizations like UN-HABITAT and WHO were reviewed. However, the Indian definitions of slums such as (i). Notified slums (as per the Slum Act 1956), (ii). Identified slums (as per the Census of India) and (iii). Recognized slums (that are not formally notified but recognized by the authorities) were not reviewed. As the study is conducted in the Indian cities of Pune and Surat, it becomes imperative to review these definitions as well besides the international definitions.
- ii. Identification of vulnerability parameters – Based on the review of both the international and national definitions, the habitat vulnerability parameters and indicators are to be identified. The number of such parameters may increase accordingly. Therefore, there is a need to augment the literature review in this regard.
- iii. Identification of indicators - Under ‘congestion’, two indicators ‘Population’ and ‘Density’ were identified. However the current literature such as Namperumal et al (2022) reveals a direct link between builtup density and positive Covid cases. Therefore, it is suggested to cite the current literature and consider ‘Builtup density’ also as one of the indicators under ‘congestion’.
- iv. Cities as study area – In the abstract, it is mentioned that two Indian megacities Pune and Surat were considered as they were the worst hit during both the waves. However no adequate data or reference is cited to substantiate this. In the background, it is mentioned as ‘40% of the cases in the 2020 wave being found in the 4 major metro cities of Delhi, Mumbai, Kolkata and Chennai’. If it is so then why these cities were not considered for the study?
- v. Study at the ward level – The study was carried out at the ward level and not at the slum level, while the title mentions as vulnerability of slums. The paper does not specify the number of slums considered in each of the wards.
- vi. True values – It is not clear as to how the true values were collected/ calculated (see Tables 3 and 4). The following questions need to be answered: How many slums were considered and based on what criteria? What is the sampling type? What is the sample size? Therefore, there is a need to improve the study design to make it more technically sound.
- vii. Data –Open source data for covid 19 risk zones (such as covid dashboard of sociometrik website) are available. Official data are available from the covid dashboard of the Ministry of Health and Family Welfare, Government of India. Therefore, there is a need to refer to such source data to ensure reproducibility.
- viii. Data presentation - Figures 4 and 5 show the ward wise relation between % Slum Population and Covid 19 infection rate. However the graphs shown are not ward wise but zone wise (south, west, north, etc.). Figures 2 & 3 and Table 3 has the ward names only but not the ward numbers, while the maps show only the ward numbers (Figure 6) and not the ward names. Therefore, there is a need to improve the data presentation and analysis.
- ix. Methods – Need to clarify the criteria for using/ adopting the linear model. Relevant literature can be reviewed for adopting such models for the purpose of calculation/ analysis. Therefore, there is a need to improve the method.
- x. Basis for ranges– Need to clarify the basis for considering the ranges (> 0.20 is taken as high, 0 to 0.20 is taken as medium and less than 0 is taken as low). Is there any literature to substantiate these ranges? Therefore, there is a need improve the method and statistical analysis.
- xi. Conclusion – In the abstract, the aim is to categorize the slums into 3 levels of risk zones. However in the conclusion, the aim is mentioned as to find a method to quantify the risk exposure to pandemics in slums. Also, It is not clear on what basis the immediate and long term solutions are suggested. Therefore, the conclusions do not fully reflect the results.
- Refer https://sociometrik.shinyapps.io/covid19riskzones/ https://covid19dashboard.mohfw.gov.in/ and [1] 

Is the work clearly and accurately presented and does it cite the current literature?

Partly

If applicable, is the statistical analysis and its interpretation appropriate?

Partly

Are all the source data underlying the results available to ensure full reproducibility?

Partly

Is the study design appropriate and is the work technically sound?

Partly

Are the conclusions drawn adequately supported by the results?

Partly

Are sufficient details of methods and analysis provided to allow replication by others?

Partly

Reviewer Expertise:

Geospatial modeling for urban planning applications,  tenure security for the urban poor, and inclusive planning

I confirm that I have read this submission and believe that I have an appropriate level of expertise to confirm that it is of an acceptable scientific standard, however I have significant reservations, as outlined above.

## References

[1] : Pandemic Spread in Metropolitan Cities of India—Spatial Planning Factors. Reference source
